# In Memoriam

**DOI:** 10.21307/jofnem-2021-112

**Published:** 2022-01-13

**Authors:** J. Ole Becker

**Affiliations:** 1 Department of Nematology, University of California, Riverside, CA 92521

**Reinhold Mankau (1928–2021)**

**Figure:**
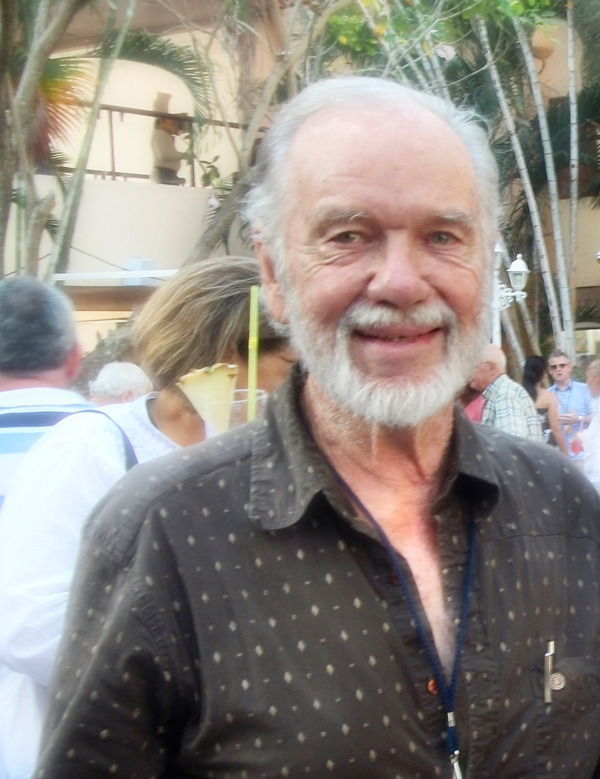


Reinhold Mankau passed away on Sunday, December 5, 2021, in Riverside, California. Known by his family, friends, and colleagues as ‘Ron’, he was a faculty member at the University of California Riverside for 33 years. He belonged to the first generation of scientists that gave the UCR Department of Nematology its stellar worldwide reputation.

Ron was born in Chicago, Illinois, on July 22, 1928. He studied Biology from 1946 to 1948 at Wright Junior College, Chicago. The following year, Ron enrolled at the University of Illinois and received a bachelor’s degree in 1951 and a Master of Science degree in 1952, both in Biology. As a Ph.D. student in Plant Pathology/Parasitology at the land-grant University of Illinois, Urbana, Plant Pathologist/Nematologist Dr. Maurice Linford was his major professor. Ron’s thesis focused on host–parasitic relationships of the clover cyst nematode, *Heterodera trifolii*. In 1954, he and the love of his life, Saroj, got married. A year later, both finished with their Ph.D. Ron received a Fulbright Research Fellowship at the Indian Agricultural Research Institute, New Delhi, India, where they lived from September 1956 to December 1957. A month later, he was appointed Assistant Nematologist at the University of California, Riverside, pursuing microbial biocontrol organisms.

In 1964–65, he spent a sabbatical as a Fulbright Senior Postdoctoral Fellow at the Agricultural College and Research Institute, Coimbatore, in southern India. In 1972, he hired Diana Wall Freckman as a Research Nematologist in his lab which started her long and exceptional career. In 1973–74, Ron served as a United Nations Development Program consultant in a project designed to advance the postgraduate program in Plant Pathology/Nematology at the University of Agricultural Science, Hebbal, Mysore, India. Consequently, he was one of the foremost experts regarding plant-parasitic nematode problems and research in India.

Ron’s research on biology and epidemiology of parasites and predators of plant-parasitic and free-living nematodes earned him a worldwide reputation as an authority in biological nematode control and sustainable crop management. Ron knew his nematode-destroying fungi like no other. His lab studied physical and biological factors that influenced their reproduction, persistence, and efficacy. His mission-oriented goal was to find microorganisms that could be efficiently and economically useful as biological control agents.

Ron also was a trailblazer in researching nematode-suppressive soils. In early 1970, he discovered a bacterium in mountainous areas of California that attached to the surface of nematodes. He found similar organisms associated with greenhouse and field populations of root-knot nematodes (*Meloidogyne* spp.) in southern California. Gerald Thorne, one of the pioneers of US Nematology, had described the microorganism previously as the microsporidian parasite *Duboscqia penetrans*. But Ron Mankau and Jack Imbriani published in 1974 after extensive study of morphology, histochemistry, and life cycle in various nematodes that the organism was a prokaryote. Ron renamed it *Bacillus penetrans* (now *Pasteuria penetrans*). However, the true potential of this and similar bacteria occurred to him while working for the French Office of Scientific and Technical Research Overseas (ORSTOM) in Senegal in 1979. He discovered *B. penetrans* parasitizing root-knot nematodes in some grower fields that significantly mitigated crop damage by those otherwise devastating pathogens. Equally important was the discovery by his Ph.D. student Graham Stirling of *Dactylella oviparasitica* (now *Hyalorbilia oviparasitica*), a hyperparasite of root-knot nematodes. They demonstrated the importance of the fungus in the natural suppression of *M. incognita* populations in some California peach orchards.

Ron taught the formal class Nematode Diseases of Plants for many years together with his Department of Nematology colleagues Ivan Thomason, Seymour Van Gundy, and Howard Ferris. He served on the Steering Committee for UCR’s Pest Management M.S. Program. He trained Ph.D. students, post-docs and inspired many more worldwide. On his and Saroj’s travels, he was often invited to give scientific seminars which he presented fluently in German, French, or Spanish. Ron was promoted to the rank of Full Professor in 1976 and retired in 1990. In Varadero, Cuba, at one of the last scientific meetings he attended, the Organization of Nematologists of Tropical America honored him with a citation of special recognition for his long-standing contribution and support of ONTA since the inception of the organization.

In retirement, he and his wife Saroj, a California State University Emeritus Professor in Biology, spent most of the year in southern Baja. Throughout their 67 years of marriage, they enjoyed traveling together, making friends on all continents, and visiting more than 100 countries around the globe. Ron was humble and personable and remained involved with the Nematology Department until shortly before his death. He will be remembered fondly by many.

